# Supplementary material for: Solvent-mediated assembly of atom-precise gold–silver nanoclusters to semiconducting one-dimensional materials
Source: Nat Commun. 2020 May 6;11:2229. doi: 10.1038/s41467-020-16062-6 (PMC7203111; doi:10.1038/s41467-020-16062-6)
Supplement: Supplementary file 1 — Supplementary Information [file 41467_2020_16062_MOESM1_ESM.pdf]

Supplementary Information for

Solvent-mediated assembly of atom-precise gold-silver nanoclusters to semiconducting one-dimensional materials

Yuan et al.

Supplementary Figures

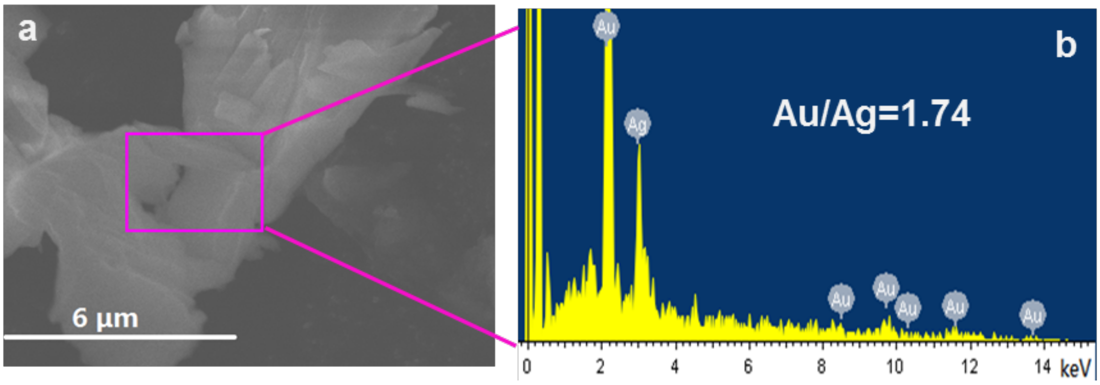

Supplementary Figure 1. (a) SEM image of **$(AuAg)_{34n}$** nanocrystals. Inset is EDS region. (b) EDS data of **$(AuAg)_{34n}$** nanocrystals.

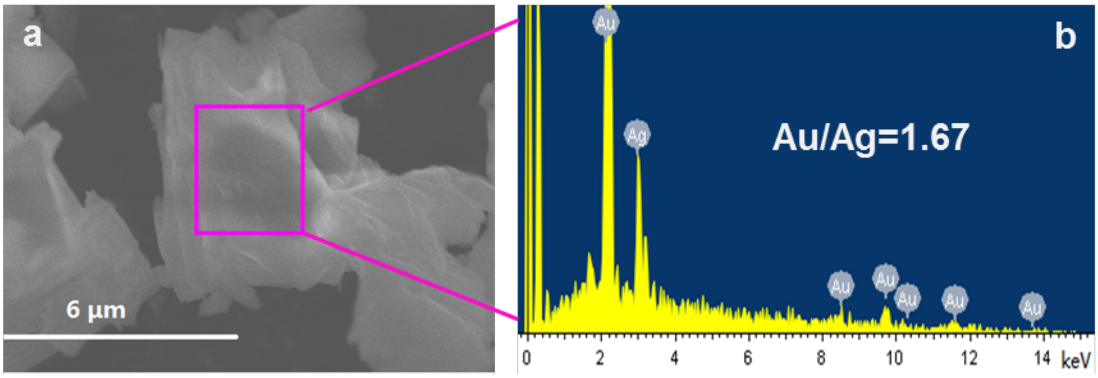

Supplementary Figure 2. (a) SEM image of **$(AuAg)_{34}$** nanocrystals. Inset is EDS region. (b) EDS data of **$(AuAg)_{34}$** nanocrystals.

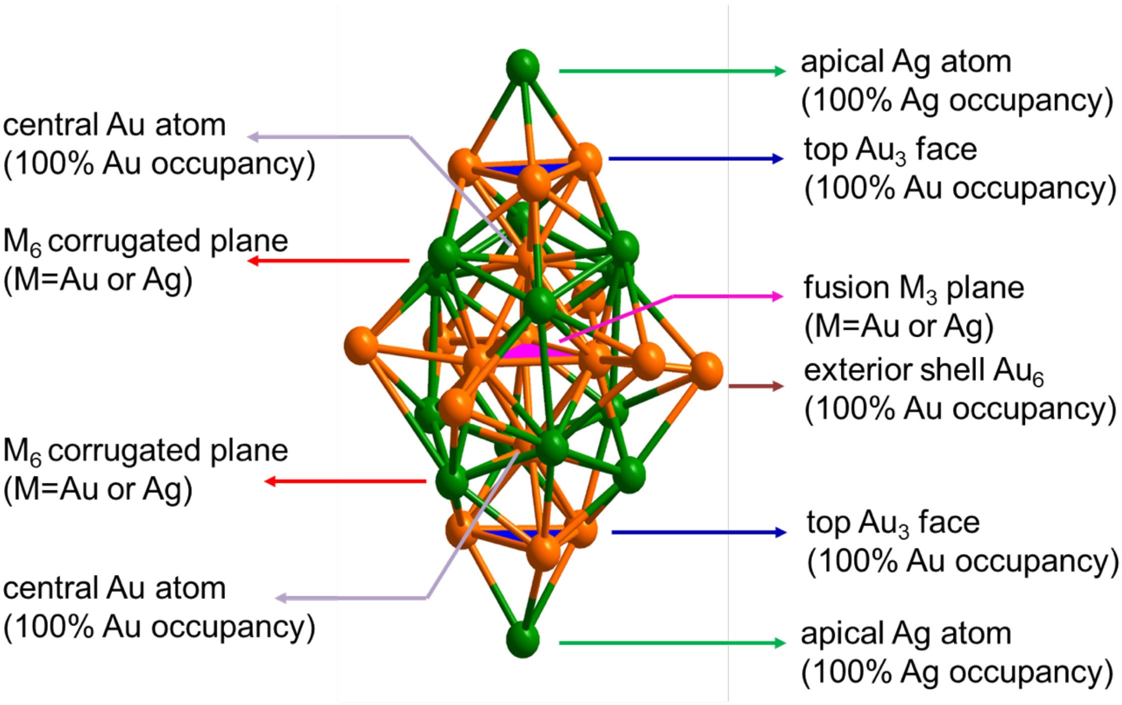

Supplementary Figure 3. The same $(AuAg)_{31}$ core structure of **$(AuAg)_{34}$** and **$(AuAg)_{34n}$** nanoclusters. Colors: golden and green, Au/Ag. Hydrogen atoms and carbon atoms are omitted for clarity.

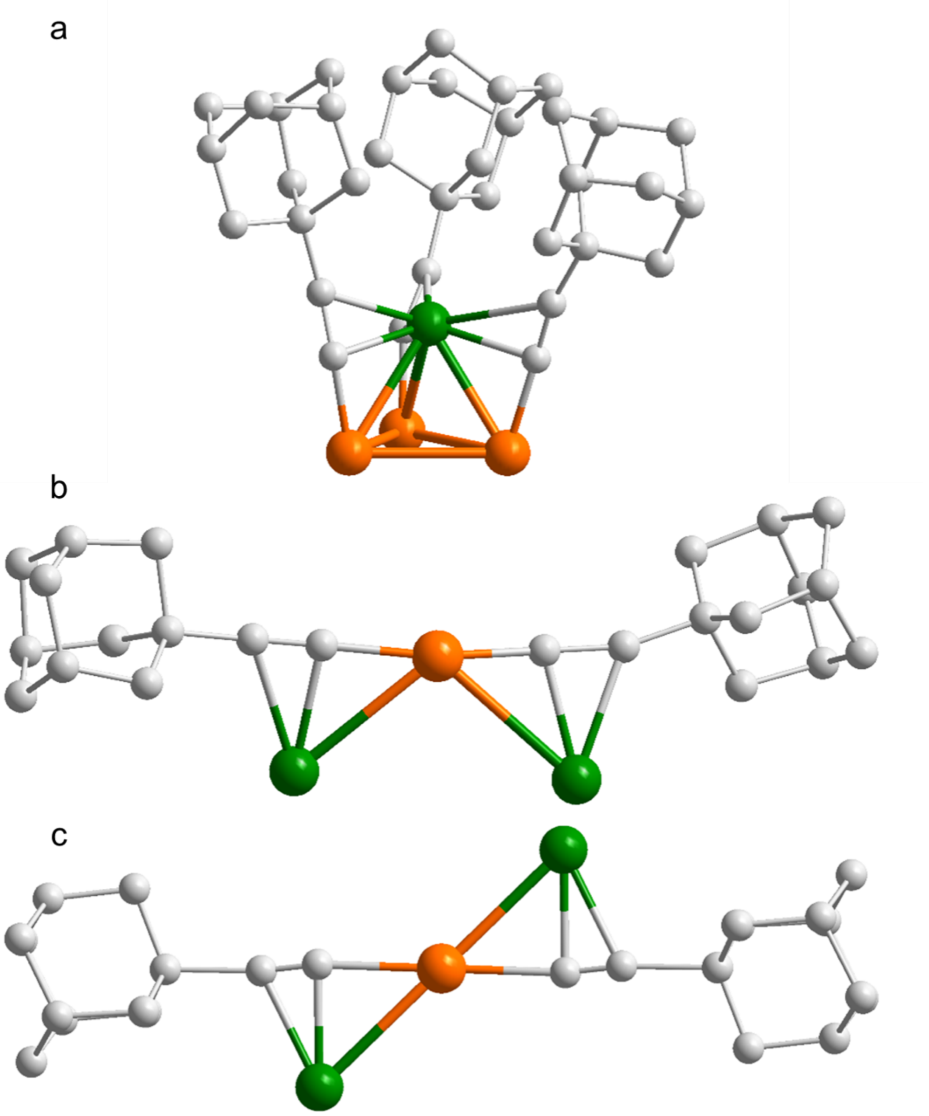

Supplementary Figure 4. The detailed bonding structure of **$(AuAg)_{34n}$** and **$(AuAg)_{34}$**. (a) The special binding structure both in **$(AuAg)_{34n}$** and **$(AuAg)_{34}$**. (b) The "linker hinge" unit "Ag-L-Au-L-Ag" in **$(AuAg)_{34}$**. (c) The "linker hinge" unit "Ag-L-Au-L-Ag" in **$(AuAg)_{34n}$**. Colors: golden, Au; green, Ag; grey, C. All hydrogen atoms are omitted for clarity.

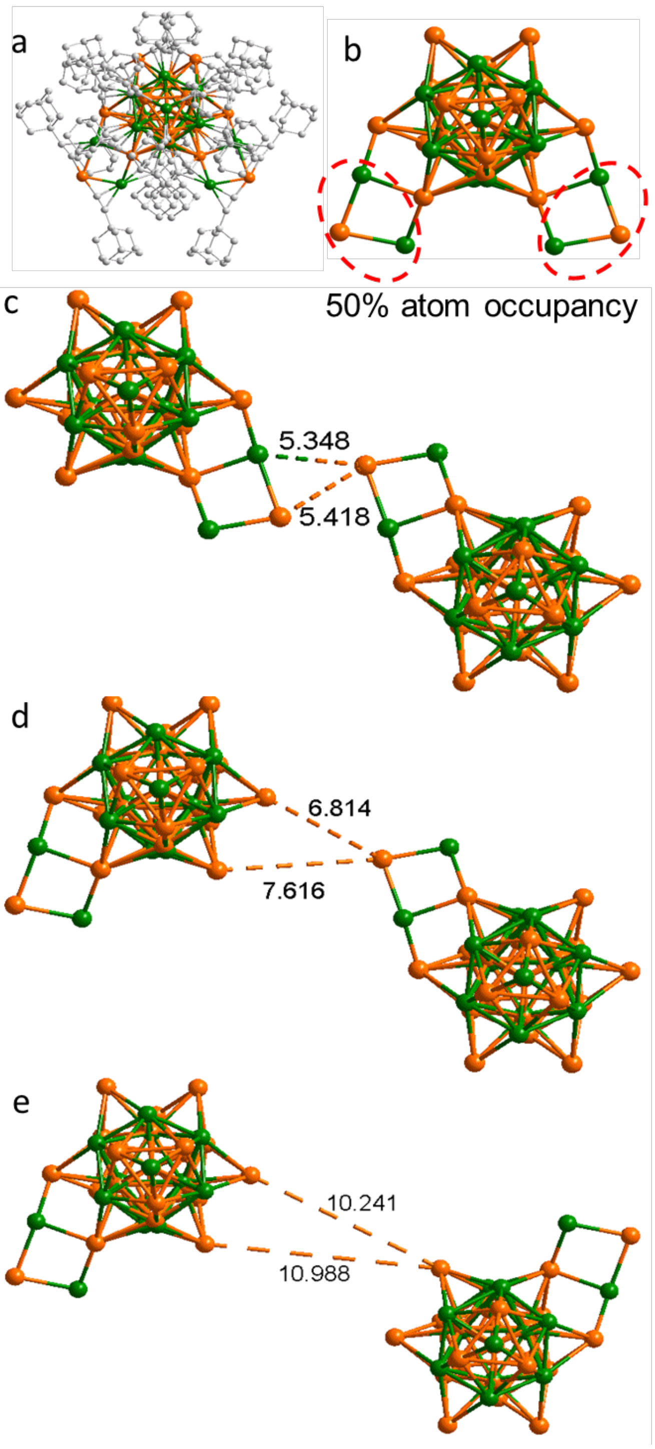

Supplementary Figure 5. (a-b) The structure of $Au_{21.3}Ag_{12.7}(Adm)_{20}$ nanocluster. (c-e) Three possibilities of the nearest adjacent nanoclusters in the real cell unit. Colors: golden and green, Au/Ag; grey, C. All hydrogen atoms are omitted for clarity.

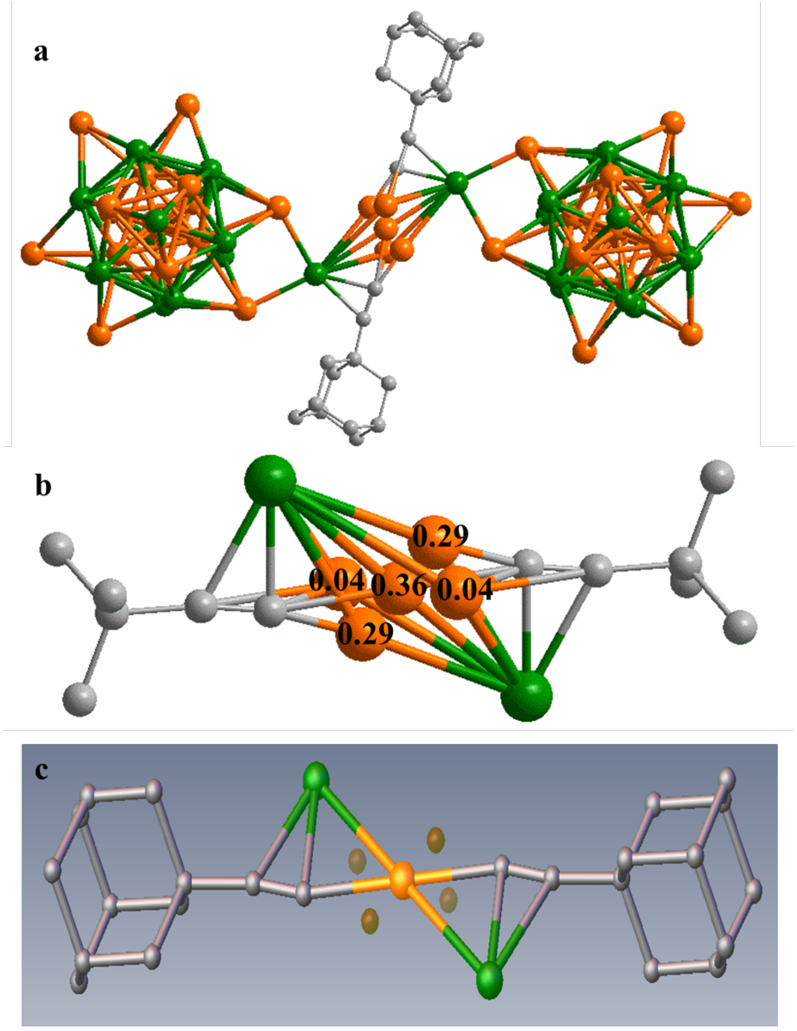

Supplementary Figure 6. The detailed Ag-Au-Ag linkage structure between $Au_{21.4}Ag_{12.6}(Adm)_{20}$ nanoclusters in the polymeric chain showing the disordering of the Au linker atom. (a) The structure shown with the neighboring clusters, (b) The detailed structure showing the arrangement and occupancies of the five possible positions of the disordered Au linker atom in the Ag-Au-Ag linkage. While the central site has an occupancy of 0.36, the other 4 sites are surrounding this central site in a nearly square geometry with occupancies of 0.29, 0.29, 0.04 and 0.04. (c) The structure of the Ag-Au-Ag linkage refined by Olex2 showing the electron densities surrounding the central Au site. Colors: golden and green, Au/Ag; grey, C; brown, the electron residual peaks. All hydrogen atoms are omitted for clarity.

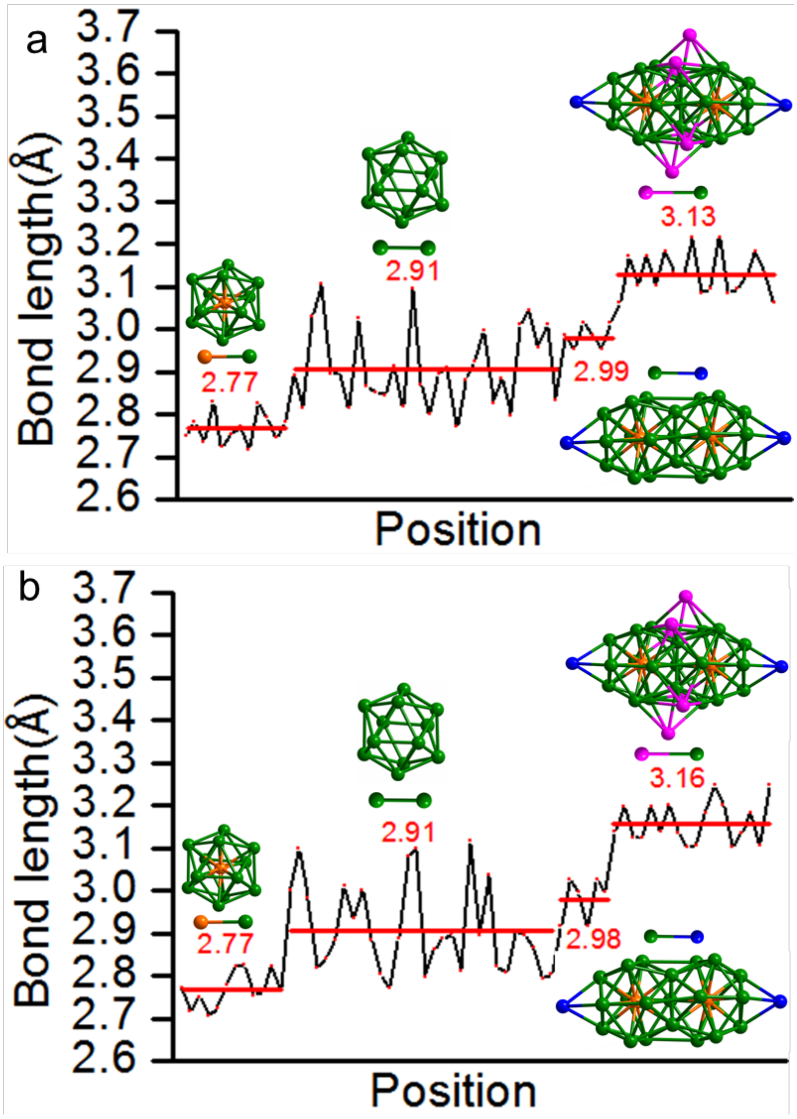

Supplementary Figure 7. Bond lengths of the same metal core structure of **$(AuAg)_{34n}$** (a) and **$(AuAg)_{34}$** (b) nanoclusters. Colors: golden, green, blue and pink, Au/Ag. All hydrogen atoms and carbon atoms are omitted for clarity.

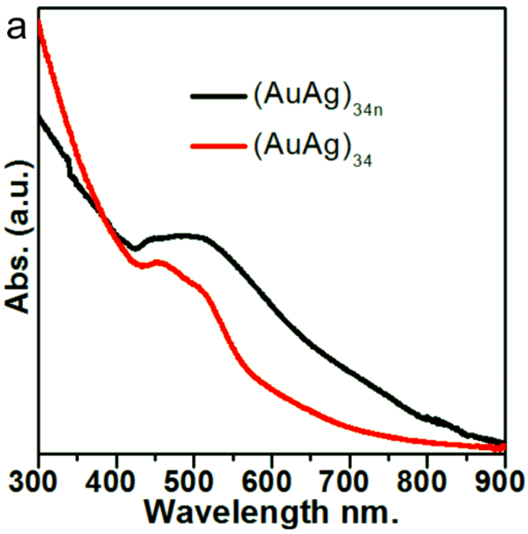

Supplementary Figure 8. UV−vis spectrum of **$(AuAg)_{34n}$** and **$(AuAg)_{34}$** in solid (wavelength x-scale).

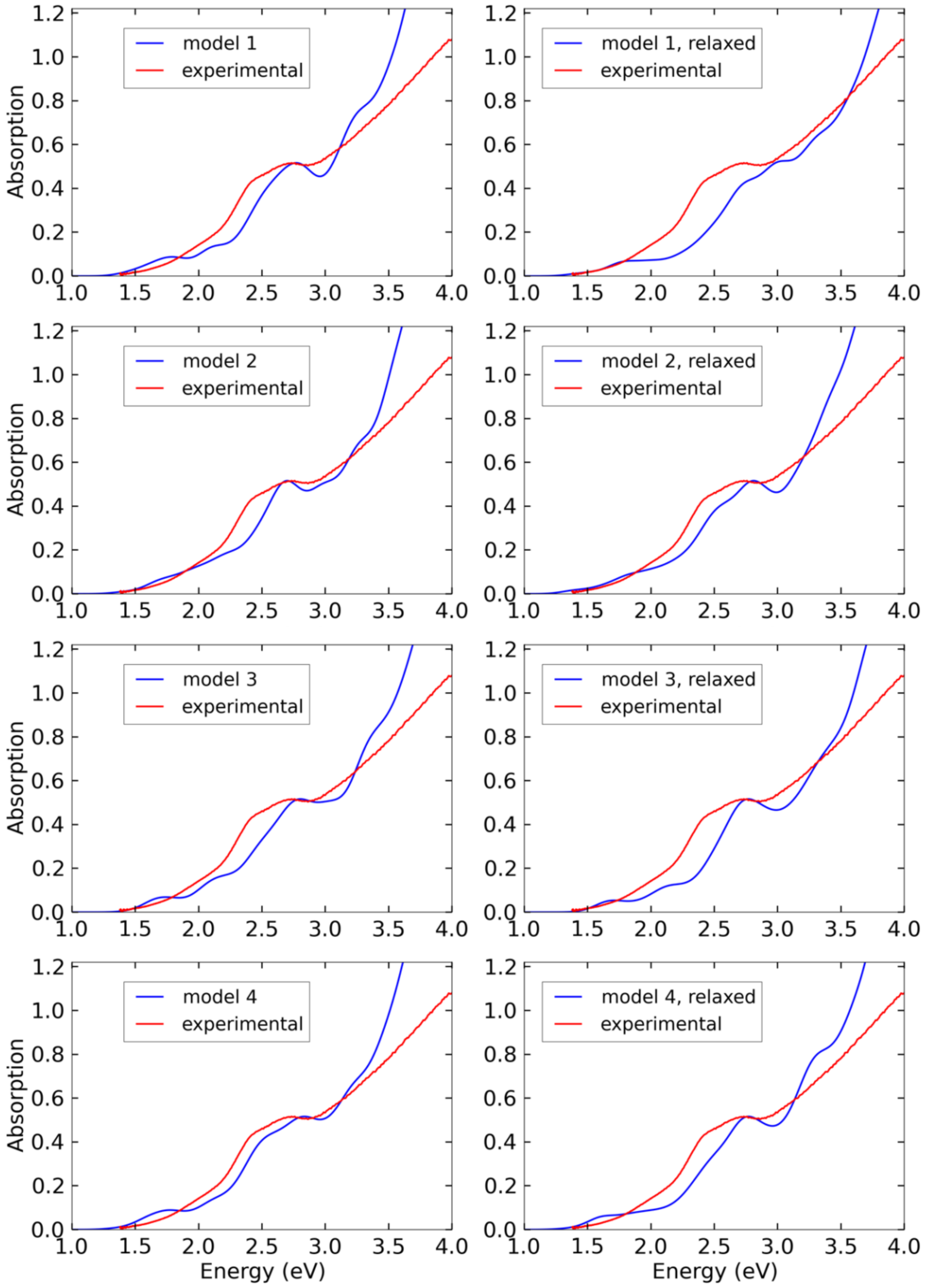

Supplementary Figure 9. Comparison of the computed and experimental uv-vis absorption spectra for **$(AuAg)_{34}$** model clusters 1-4 (see Methods). Spectra are calculated both for non-relaxed (all atom coordinates taken directly from the crystal structure) and PBE-relaxed cluster structures (atom coordinates given as Supplementary Dataset 1-4). The intensity of the computed spectra is scaled to the experimental peak at 2.7 eV but no shifts in the energy axis are applied.

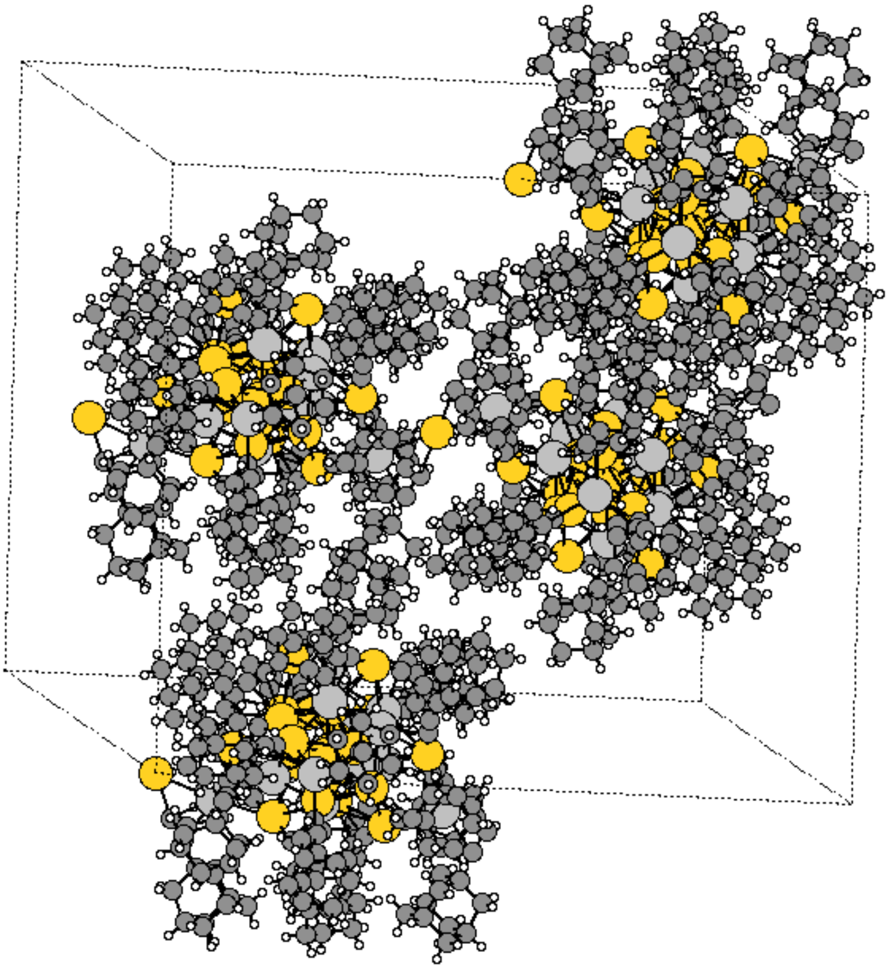

Supplementary Figure 10. The computational unit cell consisting of four $(AuAg)_{34}L_{20}$ clusters in the periodic DFT calculation of the cluster polymer crystal. The unit cell has 136 metal atoms and 80 ligand molecules. Atom coordinates are given as Supplementary Dataset 5.

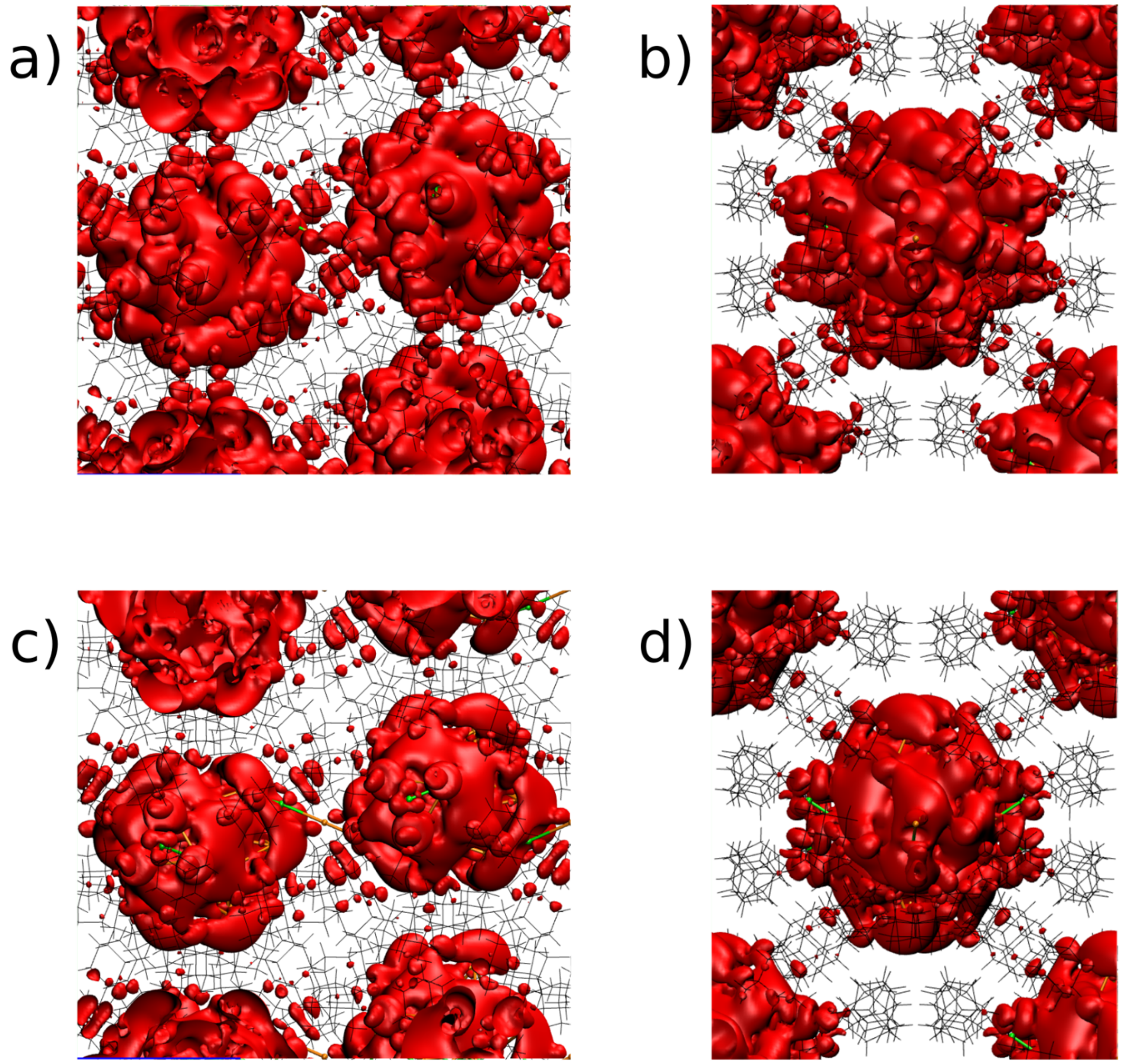

Supplementary Figure 11. Summed densities from electron states in top of the valence band (20 topmost states in a,b) and bottom of the conduction band (8 lowest states in c,d). In a) and c), the polymer axis runs horizontally whence in b) and d), the polymer axis is perpendicular to the image plane. The analysis is based on the gamma-point calculation. The isovalue surfaces are plotted by using a density of 0.0005 e/Å^3.

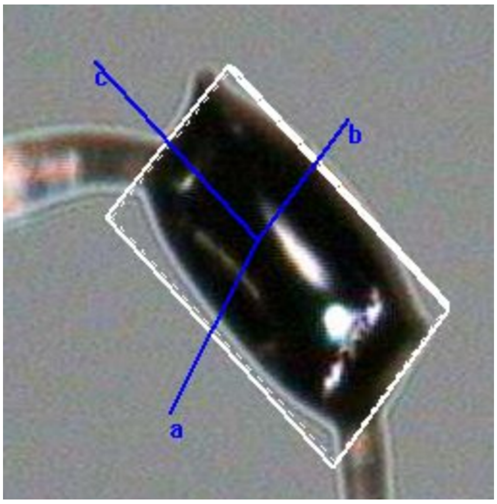

Supplementary Figure 12. A single crystal of **$(AuAg)_{34n}$** on the X-ray diffractometer and the diffractions corresponding to each face of the crystal.

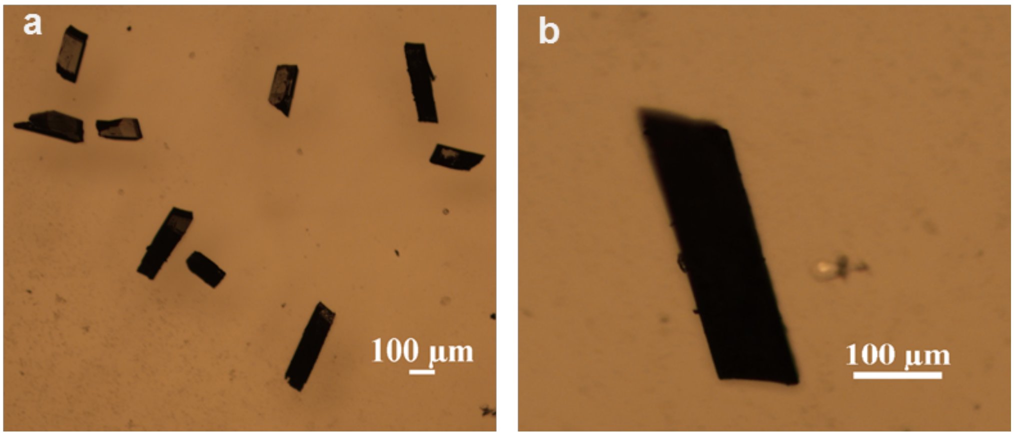

Supplementary Figure 13. (a) Optical image of **$(AuAg)_{34n}$** nanocluster crystals, (b) zoom-up image of one crystal.

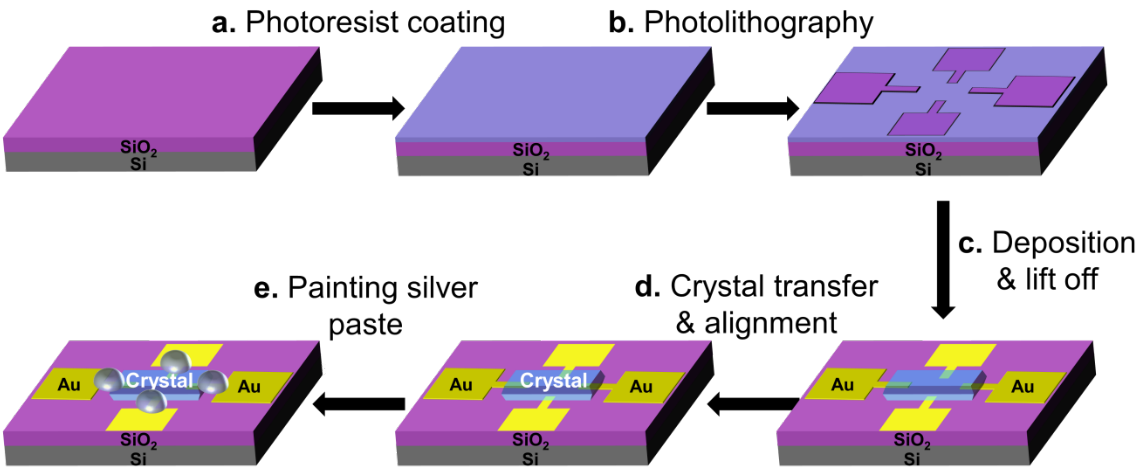

Supplementary Figure 14. Fabrication process of FET device for the measurement of electrical properties. This process includes the following steps: Step a. AZ5214E photoresists are spin coated on silicon wafer with 300 nm silicon dioxide; Step b. Metallic pattern are defined in the photoresist with photolithography; Step c. 5 nm Cr/50 nm Au are deposited with e-beam evaporation and the photoresists are lift off to obtain target metallic electrodes; Step d. Single crystal of cluster polymer are transferred onto contact with the metallic electrode and carefully align the c-crystallographic axis with electrode pairs. Step e. Silver paste was used to strengthen the contact between crystal and metallic electrode.

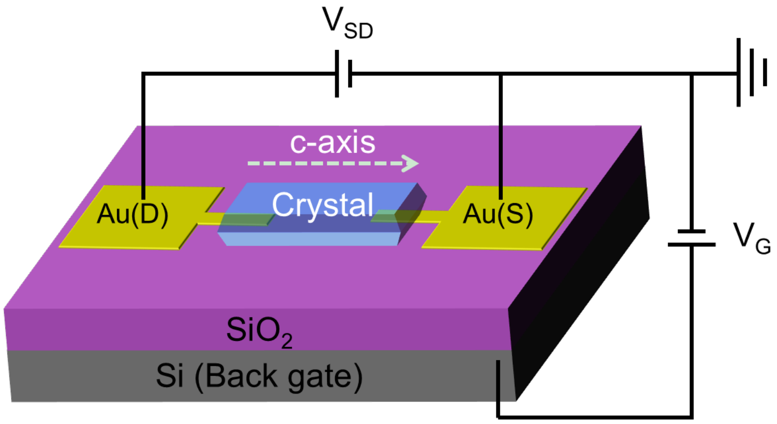

Supplementary Figure 15. Schematic illustration of the polymeric crystal FET device. Here, silicon is used as back gate and silicon oxide is used as dielectric layer. The crystal is contacted and aligned with source-drain electrode in c-crystallographic axis, acting as conducting channel.

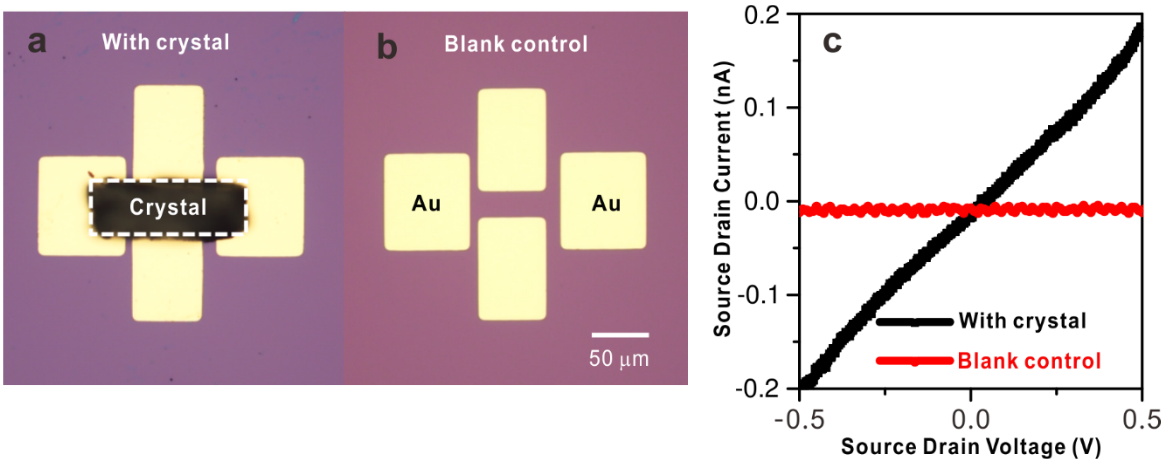

Supplementary Figure 16. (a) Optical microscope images of single crystal nanoclusters devices with and (b) blank control devices with only metal electrode but no single crystal nanoclusters transferred. (c) The conductance of single crystal-based device（black line）and blank control device (red line) measured along c-crystallographic axis at V_{SD} ranging from -0.5 to 0.5 V.

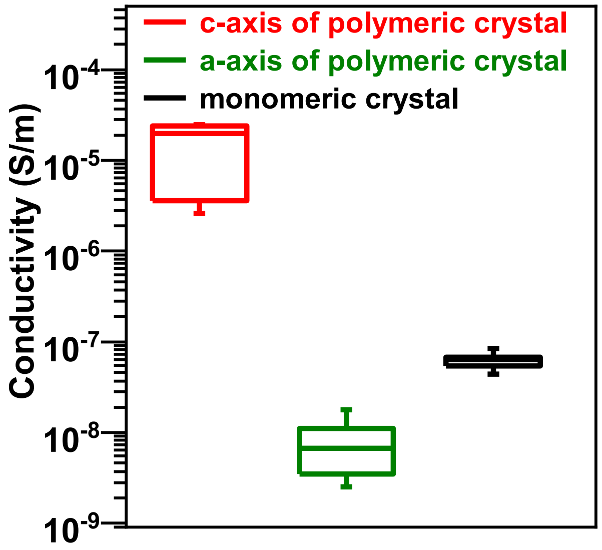

Supplementary Figure 17. Box-and-whisker plot for the conductivity of the monomeric crystal and the polymeric crystal. Shown are median, 25 and 75% quartiles, and full range.

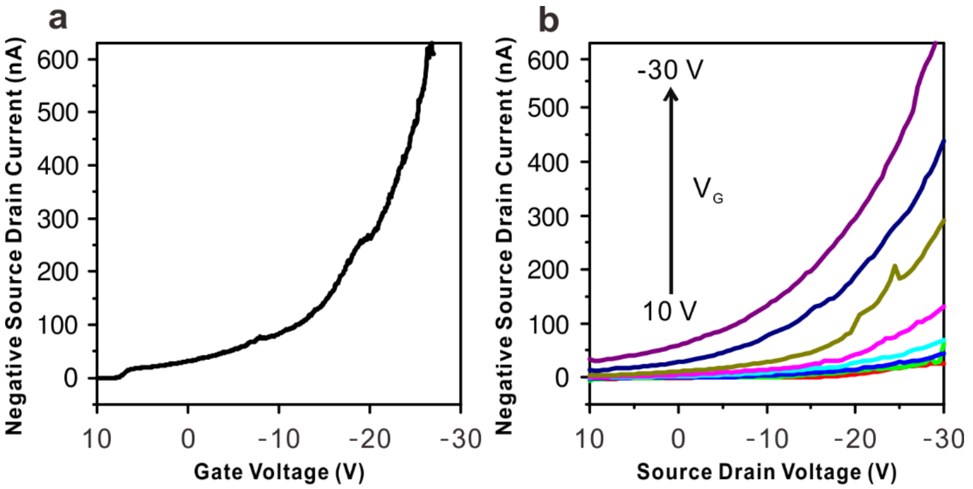

Supplementary Figure 18. Reproducibility of *p*-type semiconductor properties of polymeric crystal along c-crystallographic axis. (a) Transfer characteristics at V_{SD}= -10 V. (b) Output characteristics with V_G swept from 10 to -30 V in -5 V steps.

Supplementary Tables

Supplementary Table 1. Crystal data and structure refinement for **$(AuAg)_{34}$** and **$(AuAg)_{34n}$**

Identification code	**(AuAg)34n**	**(AuAg)34**
Empirical formula	$C_{120}H_{150}Ag_{6.28}Au_{10.74}$	$C_{240}H_{298}Ag_{12.68}Au_{21.28}$
Formula weight	4385.86	8741.24
Temperature/K	100.01(10)	100.01(10)
Crystal system	monoclinic	monoclinic
Space group	C2/c	C2/c
a/Å	27.1731(6)	40.8158(11)
b/Å	29.4239(7)	19.6669(6)
c/Å	33.2167(11)	32.0636(15)
α/°	90	90
β/°	110.913(3)	96.464(4)
γ/°	90	90
Volume/Å^3	24808.5(12)	25574.5(16)
Z	8	4
ρ_{calc}g/cm^3	2.349	2.270
μ/mm^{-1}	31.264	13.137
F(000)	16111.0	16059.0
Crystal size/mm^3	0.1 × 0.1 × 0.05	0.1 × 0.1 × 0.1
Radiation	CuKα (λ = 1.54184)	MoKα (λ = 0.71073)
2Θ range for data collection/°	6.65 to 126.052	6.558 to 54.968
Index ranges	-23 ≤ h ≤ 31, -33 ≤ k ≤ 29, -37 ≤ l ≤ 38	-52 ≤ h ≤ 51, -25 ≤ k ≤ 25, -39 ≤ l ≤ 41
Reflections collected	44694	59072
Independent reflections	19994 [R_{int} = 0.0616, R_{sigma} = 0.0619]	29050 [R_{int} = 0.0903, R_{sigma} = 0.1615]
Data/restraints/parameters	19994/1330/1316	29050/4127/1368
Goodness-of-fit on F^2	1.062	1.045
Final R indexes [I>=2σ (I)]	R_1 = 0.0801, wR_2 = 0.2156	R_1 = 0.1030, wR_2 = 0.2609
Final R indexes [all data]	R_1 = 0.1027, wR_2 = 0.2428	R_1 = 0.1908, wR_2 = 0.3419
Largest diff. peak/hole / e Å^{-3}	3.76/-4.12	7.03/-3.23

Supplementary Table 2. The occupancies of Ag/Au atoms for **$(AuAg)_{34}$**.

Sites	Ag%	Au%	Total Ag	Total Au
exterior Ag atom	100	0	2	0
central Au atoms	0	100	0	2
top Au_3 face	0	100	0	6
exterior shell Au_6	0	100	0	6
Ag(a)	50	0	1	0
the Au atom in linker	0	50	0	1
Ag(b)	50	0	1	0
M_6 corrugated plane	62.7, 67.4, 62.7, 62.7, 38.5, 60.2	37.3, 32.6, 37.3, 37.3, 61.5, 39.8	3.542	2.458
fusion M_3 plane	35.6, 35.6, 62.4,	64.4, 64.4, 37.6	1.336	1.664
M_6 corrugated plane	62.7, 67.4, 62.7, 62.7, 38.5, 60.2	37.3, 32.6, 37.3, 37.3, 61.5, 39.8	3.542	2.458
Total metal atoms			12.42	21.58
X-ray composition		**$Au_{21.3}Ag_{12.7}(A\text{-}Adm)_{20}$**		

Supplementary Table 3. The occupancies of Ag/Au atoms for **$(AuAg)_{34n}$**.

Sites	Ag%	Au%	Total Ag	Total Au
exterior Ag atom	100	0	2	0
central Au atoms	0	100	0	2
top Au_3 face	0	100	0	6
exterior shell Au_6	0	100	0	6
Ag(a)	100	0	1	0
the Au atom in linker	0	100	0	1
Ag(b)	100	0	1	0
M_6 corrugated plane	60.7	39.3	3.642	2.358
fusion M_3 plane	37.2, 37.2, 57.0	62.8, 62.8, 43.0	1.314	1.686
M_6 corrugated plane	60.7	39.3	3.642	2.358
Total metal atoms			12.60	21.40
X-ray composition		**$[Au_{21.4}Ag_{12.6}(A\text{-}Adm)_{20}]_n$**		

Supplementary Table 4. Ag/Au ratio in **$(AuAg)_{34}$** and **$(AuAg)_{34n}$** characterized by various methods.

	Method	Ag/107	Au/197	Molar (Ag)	Molar (Au)	Ag:Au molar ratio
**$(AuAg)_{34}$**	X-ray	-	-	12.7	21.3	1:1.68
	ICP-MS	41.88	130.39	0.39	0.66	1:1.69
	EDS	-	-	37.42	62.58	1:1.67
**$(AuAg)_{34n}$**	X-ray	-	-	12.6	21.4	1:1.70
	ICP-MS	50.85	164.18	0.48	0.83	1:1.73
	EDS	-	-	36.54	63.46	1:1.74

Supplementary Table 5. Electrical conductivity, the average and standard deviation of electrical conductivity along the c- and a-axis of 6 single crystals.

Devices	c-axis conductivity [S m^{-1}]	a-axis conductivity [S m^{-1}]
1	2.46×10^{-5}	8.34×10^{-9}
2	2.40×10^{-5}	1.10×10^{-8}
3	1.96×10^{-5}	1.77×10^{-8}
4	1.52×10^{-5}	6.70×10^{-9}
5	3.57×10^{-6}	3.50×10^{-9}
6	2.60×10^{-6}	2.50×10^{-9}
Averaged conductivity	1.49×10^{-5}	8.29×10^{-9}
Standard deviation	8.94×10^{-6}	5.07×10^{-9}
Anisotropic conductivity	~1800	

Supplementary Table 6. Comparison of the mobilities of our polymer crystal with traditional p-type semiconductors and super crystal of nanoparticles of n-type semiconductors.

**Semiconductor type**	**Mobility [$cm^2 V^{-1}s^{-1}$]**	**Ref**
Si	450	1
Ge	1900	1
GaAs	400	1
p-type single crystal organic semiconductor	0.002~42.7	2
p-type conducting polymers	0.00003~2.4	3
p-type small molecule organic semiconductor	0.018~5.4	3
Supercrystal of CdSe quantum Dots (n-type)	0.03	4
Supercrystals of CdSe quantum Dots (n-type)	0.005~0.1	5
Polymer crystal of nanoclusters	0.02	Our work

References

[1] Claeys, C. & Simoen, E. *Germanium-based technologies: From materials to devices*. (Elsevier, 2011).

[2] Jiang, H. & Hu, W. The emergence of organic single-crystal electronics. *Angew. Chem. Int. Ed.* **59**,1408-1428 (2020).

[3] Kumar, B., Kaushik, B. K. & Negi, Y. S. Organic thin film transistors: Structures, models, materials, fabrication, and applications: A review. *Polym Rev* **54**, 33-111 (2014).

[4] Chu, I.-H. et al. Charge transport in a quantum dot supercrystal. *J. Phys. Chem. C* **115**, 21409-21415 (2011).

[5] Talgorn, E. et al. Supercrystals of cdse quantum dots with high charge mobility and efficient electron transfer to TiO_2. *ACS Nano* **4**, 1723-1731 (2010).
