# Supplementary material for: Solvent-mediated assembly of atom-precise gold–silver nanoclusters to semiconducting one-dimensional materials
Source: Nat Commun. 2020 May 6;11:2229. doi: 10.1038/s41467-020-16062-6 (PMC7203111; doi:10.1038/s41467-020-16062-6)
Supplement: Supplementary file 3 — Description of Additional Supplementary Files [file 41467_2020_16062_MOESM3_ESM.pdf]

Description of Additional Supplementary Files

File Name: Supplementary Data 1
Description: Cluster model 1 for DFT

File Name: Supplementary Data 2
Description: Cluster model 2 for DFT

File Name: Supplementary Data 3
Description: Cluster model 3 for DFT

File Name: Supplementary Data 4
Description: Cluster model 4 for DFT

File Name: Supplementary Data 5
Description: Polymer model for DFT

File Name: Supplementary Data 6
Description: Checkcif data for AuAg34 cluster

File Name: Supplementary Data 7
Description: Checkcif data for AuAg34n polymer
