# Supplementary material for: Solvent-mediated assembly of atom-precise gold–silver nanoclusters to semiconducting one-dimensional materials
Source: Nat Commun. 2020 May 6;11:2229. doi: 10.1038/s41467-020-16062-6 (PMC7203111; doi:10.1038/s41467-020-16062-6)

checkCIF/PLATON report

You have not supplied any structure factors. As a result the full set of tests cannot be run.

THIS REPORT IS FOR GUIDANCE ONLY. IF USED AS PART OF A REVIEW PROCEDURE FOR PUBLICATION, IT SHOULD NOT REPLACE THE EXPERTISE OF AN EXPERIENCED CRYSTALLOGRAPHIC REFEREE.

No syntax errors found. CIF dictionary Interpreting this report

Datablock: yp0220170407-4-b

Bond precision: C-C = 0.0422 A Wavelength=0.71073

Cell:	a=40.8158(11)	b=19.6669(6)	c=32.0636(15)
	alpha=90	beta=96.464(4)	gamma=90
Temperature:	100 K		

	Calculated	Reported
Volume	25574.5(16)	25574.5(16)
Space group	C 2/c	C 1 2/c 1
Hall group	-C 2yc	-C 2yc
Moiety formula	C240 H298 Ag12.68 Au21.28 [+ solvent]	0.5(C480 H596 Ag25.35 Au42.56)
Sum formula	C240 H298 Ag12.68 Au21.28 [+ solvent]	C240 H298 Ag12.68 Au21.28
Mr	8741.55	8741.24
Dx,g cm-3	2.270	2.270
Z	4	4
Mu (mm-1)	13.138	13.137
F000	16059.5	16059.0
F000’	15861.82	
h,k,lmax	52,25,41	52,25,41
Nref	29365	29050
Tmin,Tmax	0.293,0.269	0.356,1.000
Tmin’	0.247	

Correction method= # Reported T Limits: Tmin=0.356 Tmax=1.000
AbsCorr = MULTI-SCAN

Data completeness= 0.989 Theta(max)= 27.484

R(reflections)= 0.1030(13401) wR2(reflections)= 0.3419(29050)

S = 1.045 Npar= 1368

The following ALERTS were generated. Each ALERT has the format
test-name_ALERT_alert-type_alert-level.
Click on the hyperlinks for more details of the test.

Alert level A

PLAT410_ALERT_2_A Short Intra H...H Contact H8B ..H2AC . 1.74 Ang.
1-x,y,1/2-z = 2_655 Check

Author Response: The two H atoms were not found on the Fourier map and belonged to different A-Adm ligands.Those linands were disorded.

PLAT410_ALERT_2_A Short Intra H...H Contact H8E ..H6GA . 1.50 Ang.
1-x,y,1/2-z = 2_655 Check

Author Response: The two H atoms were not found on the Fourier map and belonged to different A-Adm ligands.Those linands were disorded.

Alert level B

PLAT342_ALERT_3_B Low Bond Precision on C-C Bonds .... 0.04222 Ang.

Author Response: The data which arise from the ligand shell are similarly of low precision.

PLAT411_ALERT_2_B Short Inter H...H Contact H8G ..H9AA . 1.89 Ang.
1-x,-1+y,1/2-z = 2_645 Check

Author Response: The two H atoms were not found on the Fourier map and belonged to different A-Adm ligands.Those linands were disorded.

PLAT411_ALERT_2_B Short Inter H...H Contact H9BB ..H4EB . 1.95 Ang.
x,1-y,-1/2+z = 6_565 Check

Author Response: The two H atoms were not found on the Fourier map and belonged to different A-Adm ligands.Those linands were disorded.

PLAT411_ALERT_2_B Short Inter H...H Contact H7GA ..H9AB . 1.97 Ang.
1-x,-1+y,1/2-z = 2_645 Check

Author Response: The two H atoms were not found on the Fourier map and belonged to different A-Adm ligands.Those linands were disorded.

PLAT990_ALERT_1_B Deprecated .res/.hkl Input Style SQUEEZE Job ... ! Note

---

Alert level C

DIFMX02_ALERT_1_C The maximum difference density is > 0.1*ZMAX*0.75
The relevant atom site should be identified.
PLAT026_ALERT_3_C Ratio Observed / Unique Reflections (too) Low .. 46% Check
PLAT084_ALERT_3_C High wR2 Value (i.e. > 0.25) .... 0.34 Report

PLAT094_ALERT_2_C Ratio of Maximum / Minimum Residual Density ....   2.18 Report
PLAT097_ALERT_2_C Large Reported Max. (Positive) Residual Density   7.03 eA-3
PLAT213_ALERT_2_C Atom C6B   has ADP max/min Ratio .....   3.2 oblate
PLAT220_ALERT_2_C Non-Solvent Resd 1 C   Ueq(max)/Ueq(min) Range   3.9 Ratio
PLAT220_ALERT_2_C Non-Solvent Resd 1 Au   Ueq(max)/Ueq(min) Range   3.6 Ratio
PLAT241_ALERT_2_C High   ’MainMol’ Ueq as Compared to Neighbors of   C00R Check
PLAT241_ALERT_2_C High   ’MainMol’ Ueq as Compared to Neighbors of   C2B Check
PLAT241_ALERT_2_C High   ’MainMol’ Ueq as Compared to Neighbors of   C2D Check
PLAT241_ALERT_2_C High   ’MainMol’ Ueq as Compared to Neighbors of   C4D Check
PLAT241_ALERT_2_C High   ’MainMol’ Ueq as Compared to Neighbors of   C4G Check
PLAT241_ALERT_2_C High   ’MainMol’ Ueq as Compared to Neighbors of   C011 Check
PLAT241_ALERT_2_C High   ’MainMol’ Ueq as Compared to Neighbors of   C6B Check
PLAT241_ALERT_2_C High   ’MainMol’ Ueq as Compared to Neighbors of   C6D Check
PLAT241_ALERT_2_C High   ’MainMol’ Ueq as Compared to Neighbors of   C6F Check
PLAT241_ALERT_2_C High   ’MainMol’ Ueq as Compared to Neighbors of   C6G Check
PLAT241_ALERT_2_C High   ’MainMol’ Ueq as Compared to Neighbors of   C7B Check
PLAT241_ALERT_2_C High   ’MainMol’ Ueq as Compared to Neighbors of   C7F Check
PLAT241_ALERT_2_C High   ’MainMol’ Ueq as Compared to Neighbors of   C7G Check
PLAT241_ALERT_2_C High   ’MainMol’ Ueq as Compared to Neighbors of   C9G Check
PLAT241_ALERT_2_C High   ’MainMol’ Ueq as Compared to Neighbors of   C10B Check
PLAT241_ALERT_2_C High   ’MainMol’ Ueq as Compared to Neighbors of   C10D Check
PLAT241_ALERT_2_C High   ’MainMol’ Ueq as Compared to Neighbors of   C11B Check
PLAT241_ALERT_2_C High   ’MainMol’ Ueq as Compared to Neighbors of   C11D Check
PLAT241_ALERT_2_C High   ’MainMol’ Ueq as Compared to Neighbors of   C11F Check
PLAT241_ALERT_2_C High   ’MainMol’ Ueq as Compared to Neighbors of   C01L Check
PLAT241_ALERT_2_C High   ’MainMol’ Ueq as Compared to Neighbors of   C01Q Check
PLAT241_ALERT_2_C High   ’MainMol’ Ueq as Compared to Neighbors of   C33 Check
PLAT241_ALERT_2_C High   ’MainMol’ Ueq as Compared to Neighbors of   C6BA Check
PLAT241_ALERT_2_C High   ’MainMol’ Ueq as Compared to Neighbors of   C48 Check
PLAT242_ALERT_2_C Low   ’MainMol’ Ueq as Compared to Neighbors of   C1B Check
PLAT242_ALERT_2_C Low   ’MainMol’ Ueq as Compared to Neighbors of   C1D Check
PLAT242_ALERT_2_C Low   ’MainMol’ Ueq as Compared to Neighbors of   C1F Check
PLAT242_ALERT_2_C Low   ’MainMol’ Ueq as Compared to Neighbors of   C3G Check
PLAT242_ALERT_2_C Low   ’MainMol’ Ueq as Compared to Neighbors of   C5B Check
PLAT242_ALERT_2_C Low   ’MainMol’ Ueq as Compared to Neighbors of   C5D Check
PLAT242_ALERT_2_C Low   ’MainMol’ Ueq as Compared to Neighbors of   C11G Check
PLAT242_ALERT_2_C Low   ’MainMol’ Ueq as Compared to Neighbors of   C01J Check
PLAT242_ALERT_2_C Low   ’MainMol’ Ueq as Compared to Neighbors of   C6AA Check
PLAT360_ALERT_2_C Short C(sp3)-C(sp3) Bond C01A   - C01N   .   1.38 Ang.
PLAT360_ALERT_2_C Short C(sp3)-C(sp3) Bond C7BA   - C50   .   1.41 Ang.
PLAT361_ALERT_2_C Long C(sp3)-C(sp3) Bond C6AA   - C48   ..   1.67 Ang.
PLAT410_ALERT_2_C Short Intra H...H Contact H3F   ..H7CB   .   1.96 Ang.
x,y,z =   1_555 Check

Author Response: The two H atoms were not found on the Fourier map and belonged to different A-Adm ligands.Those linands were disorded.

PLAT410_ALERT_2_C Short Intra H...H Contact H33A   ..H7BD   .   1.98 Ang.
x,y,z =   1_555 Check

Author Response: The two H atoms were not found on the Fourier map and belonged to different A-Adm ligands.Those linands were disorded.

PLAT410_ALERT_2_C Short Intra H...H Contact H34A   ..H6BB   .   1.92 Ang.
x,y,z =   1_555 Check

Author Response: The two H atoms were not found on the Fourier map and belonged to different A-Adm ligands.Those linands were disorded.

```
PLAT411_ALERT_2_C Short Inter H...H Contact  H10I     ..H42A     .       2.00 Ang.
                                               x,-y,-1/2+z  =      6_555 Check
```

Author Response: The two H atoms were not found on the Fourier map and belonged to different A-Adm ligands.Those linands were disorded.

```
PLAT411_ALERT_2_C Short Inter H...H Contact  H10M     ..H6BA     .       2.07 Ang.
                                             1-x,1+y,1/2-z  =      2_665 Check
```

Author Response: The two H atoms were not found on the Fourier map and belonged to different A-Adm ligands.Those linands were disorded.

Alert level G

```
PLAT002_ALERT_2_G Number of Distance or Angle Restraints on AtSite        90 Note
PLAT003_ALERT_2_G Number of Uiso or Uij Restrained non-H Atoms ...       132 Report
PLAT005_ALERT_5_G No Embedded Refinement Details Found  in the CIF     Please Do !
PLAT042_ALERT_1_G Calc. and Reported MoietyFormula Strings  Differ     Please Check
PLAT068_ALERT_1_G Reported F000 Differs from Calcd (or Missing)...     Please Check
PLAT072_ALERT_2_G SHELXL First  Parameter in WGHT  Unusually Large      0.15 Report
PLAT083_ALERT_2_G SHELXL Second Parameter in WGHT  Unusually Large   1069.52 Why ?
PLAT300_ALERT_4_G Atom Site Occupancy of Au15       Constrained at       0.5 Check
PLAT300_ALERT_4_G Atom Site Occupancy of Au1A       Constrained at    0.3729 Check
PLAT300_ALERT_4_G Atom Site Occupancy of Au3A       Constrained at    0.3729 Check
PLAT300_ALERT_4_G Atom Site Occupancy of Au0F       Constrained at     0.326 Check
PLAT300_ALERT_4_G Atom Site Occupancy of Au0A       Constrained at    0.3729 Check
PLAT300_ALERT_4_G Atom Site Occupancy of Ag1        Constrained at       0.5 Check
PLAT300_ALERT_4_G Atom Site Occupancy of Ag05       Constrained at    0.6271 Check
PLAT300_ALERT_4_G Atom Site Occupancy of Ag06       Constrained at    0.6271 Check
PLAT300_ALERT_4_G Atom Site Occupancy of Ag4        Constrained at       0.5 Check
PLAT300_ALERT_4_G Atom Site Occupancy of Ag0D       Constrained at    0.6271 Check
PLAT300_ALERT_4_G Atom Site Occupancy of Ag0E       Constrained at     0.674 Check
PLAT300_ALERT_4_G Atom Site Occupancy of C1A        Constrained at       0.5 Check
PLAT300_ALERT_4_G Atom Site Occupancy of C1E        Constrained at       0.5 Check
PLAT300_ALERT_4_G Atom Site Occupancy of C2A        Constrained at       0.5 Check
PLAT300_ALERT_4_G Atom Site Occupancy of C2E        Constrained at       0.5 Check
PLAT300_ALERT_4_G Atom Site Occupancy of C3A        Constrained at       0.5 Check
PLAT300_ALERT_4_G Atom Site Occupancy of C3E        Constrained at       0.5 Check
PLAT300_ALERT_4_G Atom Site Occupancy of C4A        Constrained at       0.5 Check
PLAT300_ALERT_4_G Atom Site Occupancy of C4E        Constrained at       0.5 Check
PLAT300_ALERT_4_G Atom Site Occupancy of C5A        Constrained at       0.5 Check
PLAT300_ALERT_4_G Atom Site Occupancy of C5E        Constrained at       0.5 Check
PLAT300_ALERT_4_G Atom Site Occupancy of C6A        Constrained at       0.5 Check
PLAT300_ALERT_4_G Atom Site Occupancy of C6E        Constrained at       0.5 Check
PLAT300_ALERT_4_G Atom Site Occupancy of C7A        Constrained at       0.5 Check
PLAT300_ALERT_4_G Atom Site Occupancy of C7E        Constrained at       0.5 Check
PLAT300_ALERT_4_G Atom Site Occupancy of C8A        Constrained at       0.5 Check
PLAT300_ALERT_4_G Atom Site Occupancy of C8E        Constrained at       0.5 Check
PLAT300_ALERT_4_G Atom Site Occupancy of C9A        Constrained at       0.5 Check
PLAT300_ALERT_4_G Atom Site Occupancy of C9E        Constrained at       0.5 Check
PLAT300_ALERT_4_G Atom Site Occupancy of C10A       Constrained at       0.5 Check
PLAT300_ALERT_4_G Atom Site Occupancy of C10E       Constrained at       0.5 Check
PLAT300_ALERT_4_G Atom Site Occupancy of C11A       Constrained at       0.5 Check
```

```
PLAT300_ALERT_4_G Atom Site Occupancy of C11E       Constrained at        0.5 Check
PLAT300_ALERT_4_G Atom Site Occupancy of C12A       Constrained at        0.5 Check
PLAT300_ALERT_4_G Atom Site Occupancy of C12E       Constrained at        0.5 Check
PLAT300_ALERT_4_G Atom Site Occupancy of H3A        Constrained at        0.5 Check
PLAT300_ALERT_4_G Atom Site Occupancy of H3E        Constrained at        0.5 Check
PLAT300_ALERT_4_G Atom Site Occupancy of H5A        Constrained at        0.5 Check
PLAT300_ALERT_4_G Atom Site Occupancy of H5E        Constrained at        0.5 Check
PLAT300_ALERT_4_G Atom Site Occupancy of H8A        Constrained at        0.5 Check
PLAT300_ALERT_4_G Atom Site Occupancy of H8E        Constrained at        0.5 Check
PLAT300_ALERT_4_G Atom Site Occupancy of H10K       Constrained at        0.5 Check
PLAT300_ALERT_4_G Atom Site Occupancy of H10L       Constrained at        0.5 Check
PLAT300_ALERT_4_G Atom Site Occupancy of H10M       Constrained at        0.5 Check
PLAT300_ALERT_4_G Atom Site Occupancy of H10N       Constrained at        0.5 Check
PLAT300_ALERT_4_G Atom Site Occupancy of H6EA       Constrained at        0.5 Check
PLAT300_ALERT_4_G Atom Site Occupancy of H6EB       Constrained at        0.5 Check
PLAT300_ALERT_4_G Atom Site Occupancy of H7EA       Constrained at        0.5 Check
PLAT300_ALERT_4_G Atom Site Occupancy of H7EB       Constrained at        0.5 Check
PLAT300_ALERT_4_G Atom Site Occupancy of H4EA       Constrained at        0.5 Check
PLAT300_ALERT_4_G Atom Site Occupancy of H4EB       Constrained at        0.5 Check
PLAT300_ALERT_4_G Atom Site Occupancy of H2EA       Constrained at        0.5 Check
PLAT300_ALERT_4_G Atom Site Occupancy of H2EB       Constrained at        0.5 Check
PLAT300_ALERT_4_G Atom Site Occupancy of H6AB       Constrained at        0.5 Check
PLAT300_ALERT_4_G Atom Site Occupancy of H6AC       Constrained at        0.5 Check
PLAT300_ALERT_4_G Atom Site Occupancy of H4AB       Constrained at        0.5 Check
PLAT300_ALERT_4_G Atom Site Occupancy of H4AC       Constrained at        0.5 Check
PLAT300_ALERT_4_G Atom Site Occupancy of H2AC       Constrained at        0.5 Check
PLAT300_ALERT_4_G Atom Site Occupancy of H2AD       Constrained at        0.5 Check
PLAT300_ALERT_4_G Atom Site Occupancy of H7AA       Constrained at        0.5 Check
PLAT300_ALERT_4_G Atom Site Occupancy of H7AB       Constrained at        0.5 Check
PLAT300_ALERT_4_G Atom Site Occupancy of H9AA       Constrained at        0.5 Check
PLAT300_ALERT_4_G Atom Site Occupancy of H9AB       Constrained at        0.5 Check
PLAT301_ALERT_3_G Main Residue  Disorder ..............(Resd  1  )        16% Note
PLAT304_ALERT_4_G Non-Integer Number of Atoms in ...... Resd  1       571.96 Check
PLAT343_ALERT_2_G Unusual sp?      Angle Range in Main Residue for       C00Q Check
PLAT343_ALERT_2_G Unusual sp?      Angle Range in Main Residue for       C02F Check
PLAT367_ALERT_2_G Long?  C(sp?)-C(sp?) Bond  C1B      - C11B     .       1.56 Ang.
PLAT367_ALERT_2_G Long?  C(sp?)-C(sp?) Bond  C1D      - C11D     .       1.55 Ang.
PLAT367_ALERT_2_G Long?  C(sp?)-C(sp?) Bond  C1F      - C11F     .       1.55 Ang.
PLAT367_ALERT_2_G Long?  C(sp?)-C(sp?) Bond  C1G      - C11G     .       1.55 Ang.
PLAT367_ALERT_2_G Long?  C(sp?)-C(sp?) Bond  C01L     - C01S     .       1.67 Ang.
PLAT432_ALERT_2_G Short Inter X...Y Contact  C1A      ..C9E              2.19 Ang.
                                                1-x,1-y,1-z  =      5_666 Check
PLAT432_ALERT_2_G Short Inter X...Y Contact  C1A      ..C3E              3.01 Ang.
                                                1-x,1-y,1-z  =      5_666 Check
PLAT432_ALERT_2_G Short Inter X...Y Contact  C2A      ..C9E              2.16 Ang.
                                                1-x,1-y,1-z  =      5_666 Check
PLAT432_ALERT_2_G Short Inter X...Y Contact  C2A      ..C3E              2.45 Ang.
                                                1-x,1-y,1-z  =      5_666 Check
PLAT432_ALERT_2_G Short Inter X...Y Contact  C2A      ..C4E              2.82 Ang.
                                                1-x,1-y,1-z  =      5_666 Check
PLAT432_ALERT_2_G Short Inter X...Y Contact  C2E      ..C12A             2.70 Ang.
                                                1-x,1-y,1-z  =      5_666 Check
PLAT432_ALERT_2_G Short Inter X...Y Contact  C3E      ..C11A             2.60 Ang.
                                                1-x,1-y,1-z  =      5_666 Check
PLAT432_ALERT_2_G Short Inter X...Y Contact  C3E      ..C12A             2.60 Ang.
                                                1-x,1-y,1-z  =      5_666 Check
PLAT432_ALERT_2_G Short Inter X...Y Contact  C7A      ..C9E              2.54 Ang.
                                                1-x,1-y,1-z  =      5_666 Check
PLAT432_ALERT_2_G Short Inter X...Y Contact  C9E      ..C11A             2.02 Ang.
                                                1-x,1-y,1-z  =      5_666 Check
PLAT432_ALERT_2_G Short Inter X...Y Contact  C9E      ..C12A             2.37 Ang.
                                                1-x,1-y,1-z  =      5_666 Check
```

```
PLAT606_ALERT_4_G VERY LARGE Solvent Accessible VOID(S) in Structure        ! Info
PLAT720_ALERT_4_G Number of Unusual/Non-Standard Labels ..........        160 Note
PLAT764_ALERT_4_G Overcomplete CIF Bond List Detected (Rep/Expd) .       1.13 Ratio
PLAT773_ALERT_2_G Check long C-C Bond in CIF: C9E      --C11A            2.02 Ang.
PLAT773_ALERT_2_G Check long C-C Bond in CIF: C11A     --C9E             2.02 Ang.
PLAT793_ALERT_4_G Model has Chirality at C3E         (Centro SPGR)          R Verify
PLAT793_ALERT_4_G Model has Chirality at C8E         (Centro SPGR)          S Verify
PLAT860_ALERT_3_G Number of Least-Squares Restraints .............       4127 Note
PLAT869_ALERT_4_G ALERTS Related to the Use of SQUEEZE Suppressed           ! Info
PLAT883_ALERT_1_G No Info/Value for _atom_sites_solution_primary .     Please Do !
```

```
  2 ALERT level A = Most likely a serious problem - resolve or explain
  5 ALERT level B = A potentially serious problem, consider carefully
 49 ALERT level C = Check. Ensure it is not caused by an omission or oversight
100 ALERT level G = General information/check it is not something unexpected

  5 ALERT type 1 CIF construction/syntax error, inconsistent or missing data
 75 ALERT type 2 Indicator that the structure model may be wrong or deficient
  5 ALERT type 3 Indicator that the structure quality may be low
 70 ALERT type 4 Improvement, methodology, query or suggestion
  1 ALERT type 5 Informative message, check
```

It is advisable to attempt to resolve as many as possible of the alerts in all categories. Often the minor alerts point to easily fixed oversights, errors and omissions in your CIF or refinement strategy, so attention to these fine details can be worthwhile. In order to resolve some of the more serious problems it may be necessary to carry out additional measurements or structure refinements. However, the purpose of your study may justify the reported deviations and the more serious of these should normally be commented upon in the discussion or experimental section of a paper or in the "special_details" fields of the CIF. checkCIF was carefully designed to identify outliers and unusual parameters, but every test has its limitations and alerts that are not important in a particular case may appear. Conversely, the absence of alerts does not guarantee there are no aspects of the results needing attention. It is up to the individual to critically assess their own results and, if necessary, seek expert advice.

Publication of your CIF in IUCr journals

A basic structural check has been run on your CIF. These basic checks will be run on all CIFs submitted for publication in IUCr journals (*Acta Crystallographica*, *Journal of Applied Crystallography*, *Journal of Synchrotron Radiation*); however, if you intend to submit to *Acta Crystallographica Section C* or *E* or *IUCrData*, you should make sure that full publication checks are run on the final version of your CIF prior to submission.

Publication of your CIF in other journals

Please refer to the *Notes for Authors* of the relevant journal for any special instructions relating to CIF submission.

PLATON version of 03/05/2019; check.def file version of 29/04/2019

Datablock yp0220170407-4-b - ellipsoid plot

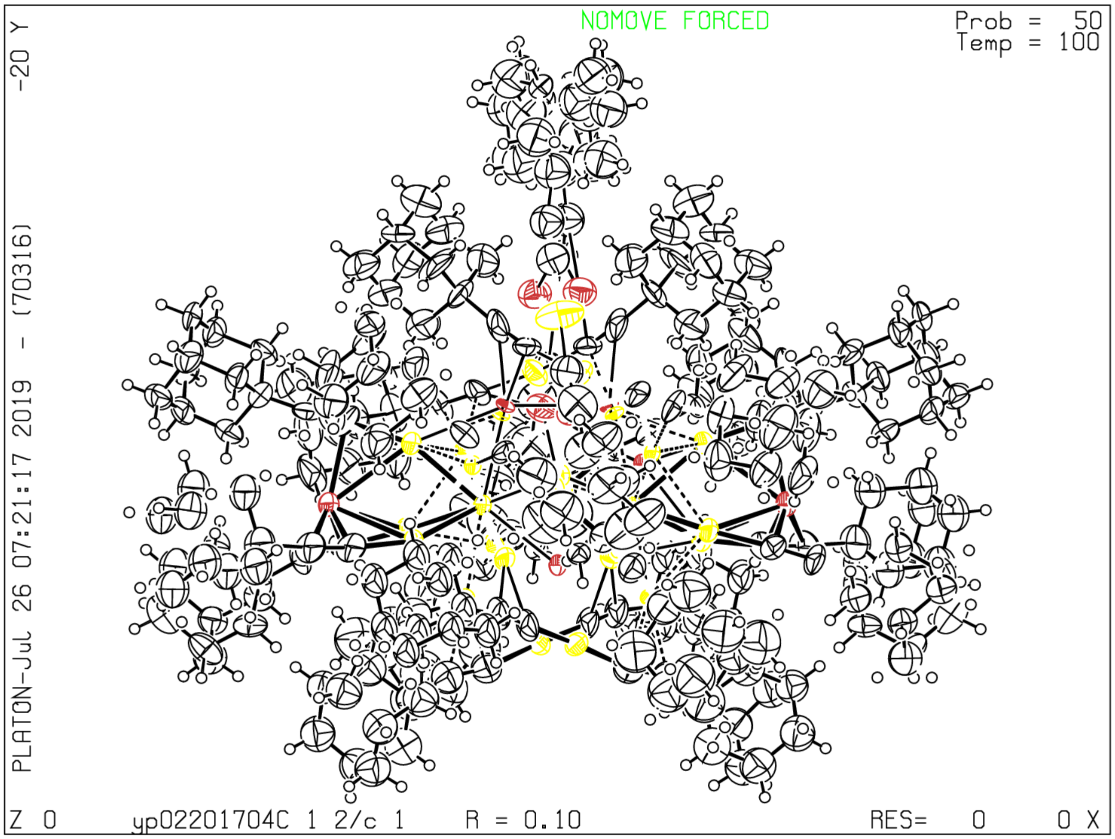

Supplement: Supplementary file 9 — Supplementary Data 6 [file 41467_2020_16062_MOESM9_ESM.pdf]
