# Supplementary material for: Solvent-mediated assembly of atom-precise gold–silver nanoclusters to semiconducting one-dimensional materials
Source: Nat Commun. 2020 May 6;11:2229. doi: 10.1038/s41467-020-16062-6 (PMC7203111; doi:10.1038/s41467-020-16062-6)

checkCIF/PLATON report

You have not supplied any structure factors. As a result the full set of tests cannot be run.

THIS REPORT IS FOR GUIDANCE ONLY. IF USED AS PART OF A REVIEW PROCEDURE FOR PUBLICATION, IT SHOULD NOT REPLACE THE EXPERTISE OF AN EXPERIENCED CRYSTALLOGRAPHIC REFEREE.

No syntax errors found. CIF dictionary Interpreting this report

Datablock: yp1161019-3-a

```
Bond precision:  C-C = 0.0449 A          Wavelength=1.54184

Cell:           a=27.1731(6)    b=29.4239(7)       c=33.2167(11)
                alpha=90        beta=110.913(3)    gamma=90
Temperature:    100 K
```

	Calculated	Reported
Volume	24808.5(13)	24808.5(12)
Space group	C 2/c	C 1 2/c 1
Hall group	-C 2yc	-C 2yc
Moiety formula	C120 H150 Ag6.26 Au10.77 [+ solvent]	C120 H150 Ag6.26 Au10.77
Sum formula	C120 H150 Ag6.26 Au10.77 [+ solvent]	C120 H150 Ag6.28 Au10.74
Mr	4387.90	4385.86
Dx,g cm-3	2.350	2.349
Z	8	8
Mu (mm-1)	31.285	31.264
F000	16116.8	16111.0
F000’	15778.53	
h,k,lmax	31,34,38	31,33,38
Nref	20076	19994
Tmin,Tmax		0.073,1.000
Tmin’		

```
Correction method= # Reported T Limits: Tmin=0.073 Tmax=1.000
AbsCorr = MULTI-SCAN

Data completeness= 0.996           Theta(max)= 63.026

R(reflections)= 0.0801( 15422)    wR2(reflections)= 0.2428( 19994)

S = 1.062                    Npar= 1316
```

```
The following ALERTS were generated. Each ALERT has the format
       test-name_ALERT_alert-type_alert-level.
Click on the hyperlinks for more details of the test.
```

Alert level B

```
PLAT342_ALERT_3_B Low Bond Precision on  C-C Bonds ...............    0.04493 Ang.
```

Author Response: The data which arise from the ligand shell are similarly of low precision.

```
PLAT990_ALERT_1_B Deprecated .res/.hkl Input Style SQUEEZE Job ...          ! Note
```

Alert level C

```
THETM01_ALERT_3_C  The value of sine(theta_max)/wavelength is less than 0.590
            Calculated sin(theta_max)/wavelength =    0.5780
PLAT018_ALERT_1_C _diffrn_measured_fraction_theta_max .NE. *_full           ! Check
PLAT053_ALERT_1_C Minimum Crystal Dimension Missing (or Error) ...     Please Check
PLAT054_ALERT_1_C Medium  Crystal Dimension Missing (or Error) ...     Please Check
PLAT055_ALERT_1_C Maximum Crystal Dimension Missing (or Error) ...     Please Check
PLAT213_ALERT_2_C Atom Au7A            has ADP max/min Ratio .....        3.8 oblate
PLAT213_ALERT_2_C Atom Ag7B            has ADP max/min Ratio .....        3.6 prolat
PLAT220_ALERT_2_C Non-Solvent  Resd 1 Ag   Ueq(max)/Ueq(min) Range        3.6 Ratio
PLAT241_ALERT_2_C High   'MainMol' Ueq as Compared to Neighbors of       Ag0A Check
PLAT241_ALERT_2_C High   'MainMol' Ueq as Compared to Neighbors of       C109 Check
PLAT242_ALERT_2_C Low    'MainMol' Ueq as Compared to Neighbors of        Au5 Check
PLAT360_ALERT_2_C Short  C(sp3)-C(sp3) Bond  C66      - C71      .       1.41 Ang.
PLAT366_ALERT_2_C Short? C(sp?)-C(sp?) Bond  C1       - C2       .       1.14 Ang.
PLAT366_ALERT_2_C Short? C(sp?)-C(sp?) Bond  C13      - C14      .       1.14 Ang.
PLAT366_ALERT_2_C Short? C(sp?)-C(sp?) Bond  C25      - C26      .       1.08 Ang.
PLAT372_ALERT_2_C Short  C(sp)-C(sp)   Bond  C37      - C38      .       1.12 Ang.
PLAT410_ALERT_2_C Short Intra H...H Contact  H29A     ..H35A     .       1.93 Ang.
                                                      x,y,z  =      1_555 Check
PLAT411_ALERT_2_C Short Inter H...H Contact  H23      ..H71      .       2.01 Ang.
                                          3/2-x,-1/2+y,3/2-z  =      4_646 Check
```

Alert level G

```
FORMU01_ALERT_1_G  There is a discrepancy between the atom counts in the
            _chemical_formula_sum and _chemical_formula_moiety. This is
            usually due to the moiety formula being in the wrong format.
            Atom count from _chemical_formula_sum:   C120 H150 Ag6.28 Au10.74
            Atom count from _chemical_formula_moiety:C120 H150 Ag6.26 Au10.77
FORMU01_ALERT_2_G  There is a discrepancy between the atom counts in the
            _chemical_formula_sum and the formula from the _atom_site* data.
            Atom count from _chemical_formula_sum:C120 H150 Ag6.28 Au10.74
            Atom count from the _atom_site data:  C120 H150 Ag6.258 Au10.76550
CELLZ01_ALERT_1_G Difference between formula and atom_site contents detected.
CELLZ01_ALERT_1_G ALERT: check formula stoichiometry or atom site occupancies.
           From the CIF: _cell_formula_units_Z    8
           From the CIF: _chemical_formula_sum  C120 H150 Ag6.28 Au10.74
           TEST: Compare cell contents of formula and atom_site data

           atom    Z*formula  cif sites diff
           C         960.00    960.00    0.00
           H        1200.00   1200.00    0.00
           Ag         50.24     50.06    0.18
           Au         85.92     86.12   -0.20
```

```
PLAT002_ALERT_2_G Number of Distance or Angle Restraints on AtSite         83 Note
PLAT003_ALERT_2_G Number of Uiso or Uij Restrained non-H Atoms ...        137 Report
PLAT004_ALERT_5_G Polymeric Structure Found with Maximum Dimension          1 Info
PLAT005_ALERT_5_G No Embedded Refinement Details Found  in the CIF     Please Do !
PLAT041_ALERT_1_G Calc. and Reported SumFormula    Strings  Differ     Please Check
PLAT068_ALERT_1_G Reported F000 Differs from Calcd (or Missing)...     Please Check
PLAT072_ALERT_2_G SHELXL First  Parameter in WGHT  Unusually Large       0.13 Report
PLAT083_ALERT_2_G SHELXL Second Parameter in WGHT  Unusually Large    1805.57 Why ?
PLAT300_ALERT_4_G Atom Site Occupancy of Au2A       Constrained at       0.43 Check
PLAT300_ALERT_4_G Atom Site Occupancy of Ag         Constrained at       0.57 Check
PLAT301_ALERT_3_G Main Residue  Disorder ..............(Resd  1  )        6% Note
PLAT343_ALERT_2_G Unusual sp?      Angle Range in Main Residue for         C1 Check
PLAT343_ALERT_2_G Unusual sp?      Angle Range in Main Residue for        C13 Check
PLAT343_ALERT_2_G Unusual sp?      Angle Range in Main Residue for        C25 Check
PLAT343_ALERT_2_G Unusual sp?      Angle Range in Main Residue for        C50 Check
PLAT343_ALERT_2_G Unusual sp?      Angle Range in Main Residue for        C85 Check
PLAT606_ALERT_4_G VERY LARGE Solvent Accessible VOID(S) in Structure        ! Info
PLAT720_ALERT_4_G Number of Unusual/Non-Standard Labels ..........          2 Note
PLAT764_ALERT_4_G Overcomplete CIF Bond List Detected (Rep/Expd) .       1.19 Ratio
PLAT779_ALERT_4_G Suspect or Irrelevant (Bond) Angle in CIF .... #        661 Check
              AU6A -C109 -AU6     1.555   1.555   1.555            24.10 Deg.
PLAT779_ALERT_4_G Suspect or Irrelevant (Bond) Angle in CIF .... #        663 Check
              AU0A -C109 -AU6     1.555   1.555   1.555            29.50 Deg.
PLAT779_ALERT_4_G Suspect or Irrelevant (Bond) Angle in CIF .... #        664 Check
              AU0A -C109 -AU6A    1.555   1.555   1.555            34.80 Deg.
PLAT860_ALERT_3_G Number of Least-Squares Restraints .............       1330 Note
PLAT869_ALERT_4_G ALERTS Related to the Use of SQUEEZE Suppressed           ! Info
PLAT883_ALERT_1_G No Info/Value for _atom_sites_solution_primary .     Please Do !
```

---

0 **ALERT level A** = Most likely a serious problem - resolve or explain
2 **ALERT level B** = A potentially serious problem, consider carefully
18 **ALERT level C** = Check. Ensure it is not caused by an omission or oversight
29 **ALERT level G** = General information/check it is not something unexpected

11 ALERT type 1 CIF construction/syntax error, inconsistent or missing data
23 ALERT type 2 Indicator that the structure model may be wrong or deficient
4 ALERT type 3 Indicator that the structure quality may be low
9 ALERT type 4 Improvement, methodology, query or suggestion
2 ALERT type 5 Informative message, check

It is advisable to attempt to resolve as many as possible of the alerts in all categories. Often the minor alerts point to easily fixed oversights, errors and omissions in your CIF or refinement strategy, so attention to these fine details can be worthwhile. In order to resolve some of the more serious problems it may be necessary to carry out additional measurements or structure refinements. However, the purpose of your study may justify the reported deviations and the more serious of these should normally be commented upon in the discussion or experimental section of a paper or in the "special_details" fields of the CIF. checkCIF was carefully designed to identify outliers and unusual parameters, but every test has its limitations and alerts that are not important in a particular case may appear. Conversely, the absence of alerts does not guarantee there are no aspects of the results needing attention. It is up to the individual to critically assess their own results and, if necessary, seek expert advice.

Publication of your CIF in IUCr journals

A basic structural check has been run on your CIF. These basic checks will be run on all CIFs submitted for publication in IUCr journals (*Acta Crystallographica*, *Journal of Applied Crystallography*, *Journal of Synchrotron Radiation*); however, if you intend to submit to *Acta Crystallographica Section C* or *E* or *IUCrData*, you should make sure that full publication checks are run on the final version of your CIF prior to submission.

Publication of your CIF in other journals

Please refer to the *Notes for Authors* of the relevant journal for any special instructions relating to CIF submission.

PLATON version of 03/05/2019; check.def file version of 29/04/2019

Datablock yp1161019-3-a - ellipsoid plot

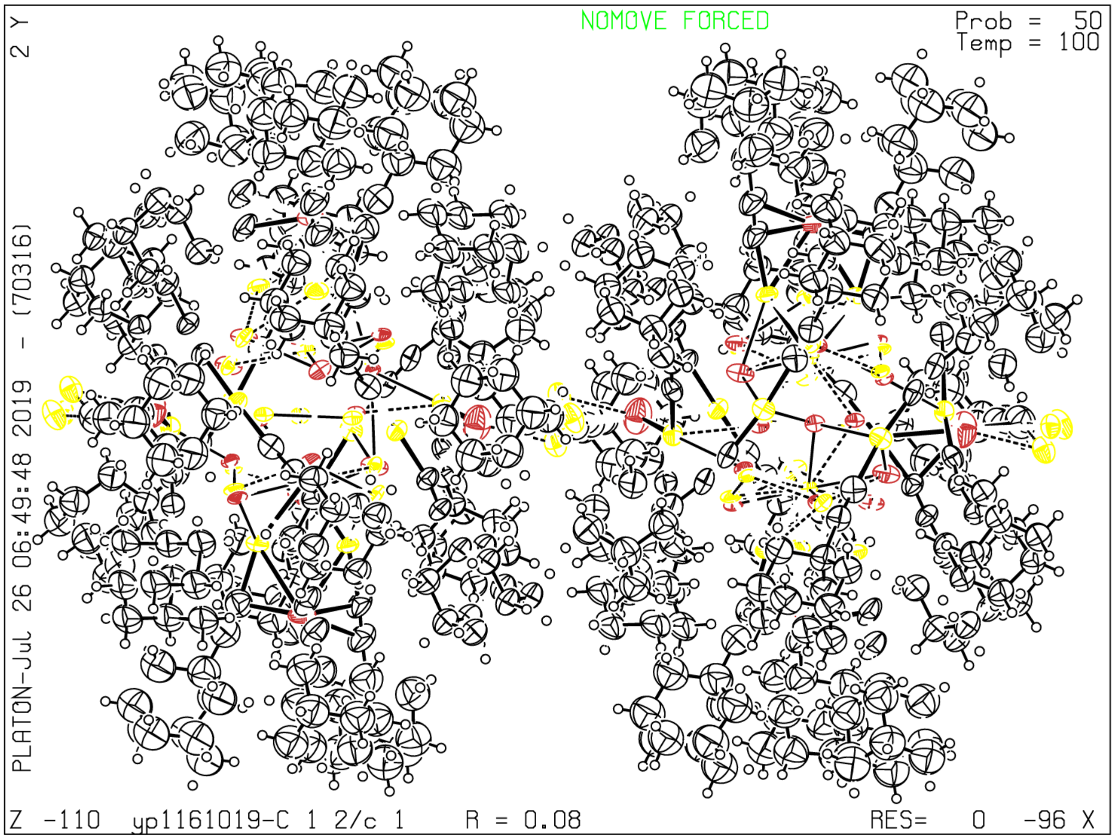

Supplement: Supplementary file 10 — Supplementary Data 7 [file 41467_2020_16062_MOESM10_ESM.pdf]
